# Items

**Published:** 1886-03

**Authors:** 


### ITEMS.

---

Minister's wife (looking over the paper): "You are referred to in this morning's paper, my dear, as a distinguished clergyman."

Minister: "H'm! I thought that my sermon yesterday would attract attention. Is it published in full, or only a synopsis given?"

Wife: "Neither; you are spoken of as a 'distinguished clergyman' in connection with that patent medicine testimonial you sent Dr. Quack."

